# Necrotizing fasciitis caused by the treatment of chronic non-specific back pain

**DOI:** 10.1186/s12871-020-01161-0

**Published:** 2020-09-26

**Authors:** Lilit Floether, Michael Bucher, Ralf Benndorf, Anna-Maria Burgdorff

**Affiliations:** 1 Department of Anesthesiology and Surgical Intensive Care, University Hospital Halle (Saale), Ernst-Grube-Straße 40, 06120 Halle (Saale), Germany; 2 Department of Clinical Pharmacy and Pharmacotherapy, Martin Luther University Halle-Wittenberg, Halle (Saale), Germany

**Keywords:** Chronic back pain, Necrotizing fasciitis, Hyperbaric oxygenation, Infiltration

## Abstract

**Background:**

Chronic back pain is a multifactorial disease that occurs particularly in adults and has many negative effects on the quality of daily life. Therapeutic strategies are often multimodal and designed for a long-term therapy period. In some cases, one option is joint infiltration or intrathecal injection with local anaesthetics. An adverse effect of this intervention may be necrotic fasciitis, a disease with high mortality and few therapeutic options.

**Case presentation:**

This case shows a 53-year-old female patient who developed necrotic fasciitis after infiltrations of the sacroiliac joint and after epidural-sacral and intrathecal injections.

**Conclusion:**

Thanks to early and aggressive surgical intervention, antibiotic treatment and hyperbaric oxygenation, she survived this serious complication and was able to return to life.

## Background

Chronic back pain is a worldwide disease that affects 70–80% of all adults during their life. Due to the duration and persistence of pain, it is associated with a significant disability in everyday life as well as a high psychosocial burden. This leads to high health care costs, absenteeism and economic burden [1, 2].

Due to the complexity of chronic non-specific back pain, curative therapy usually consists of a multimodal concept. In the national German guidelines for the treatment of non-specific back pain, various non-drug measures (exercise therapy, acupuncture, psychological care) and drug measures (non-opioid analgesics, opioids) are recommended, whereby no recommendation could be given for invasive or intramuscular (subcutaneous) application [3]. Similarly, the European Guidelines for management of chronic nonspecific low back pain do not recommend epidural corticosteroids, intra-articular (facet) steroid injections and some other invasive treatments [4]. A necrotizing fasciitis may be a possible, albeit very rare, complication of such an invasive procedure. Causes of necrotizing fasciitis are usually bacterial infections (often beta-hemolytic Group A Streptococci or mixed infections) through injuries to the skin, e.g. punctures or perforations. Risk factors are diseases that often lead to microtrauma of the skin or wound infections, such as peripheral arterial disease, diabetes mellitus or obesity. Its course is characterized by a rapid progression and a high mortality rate of about 20% [5]. The therapeutic strategies of necrotizing fasciitis include early surgical intervention, antibiotic therapy and adjuvant measures such as hyperbaric oxygenation [6]. In this context, HBO, as part of a multimodal strategy consisting of surgery, antibiotics and intensive care, may reduce mortality from 34 to 11.9% compared to standard care [7].

The following casuistry describes a 53-year-old female patient who developed a fulminant necrotizing fasciitis after infiltration therapy for chronic non-specific back pain.

## Case presentation

The 53-year-old patient came to our department as an acute transfer via our central emergency room and presented with clinical symptoms of necrotizing fasciitis.

For more than 6 years, the patient has been complaining of recurrent pain in the lumbar spine with radiation into the right lower leg but without a sensorimotor deficit. In the past years she has presented several times as an outpatient and inpatient for pain therapy, where she received facet joint infiltrations and epidural-sacral injections, which provided her with short-term pain relief. Before her transfer to our clinic, the patient had received infiltrations of the sacroiliac joint (ISG) on January 14 and 18 with bupivacaine 0.5% and dexmethasone 4 mg, epidural-sacral on January 15 and 17 with prilocaine 1%, bupivacaine 0.5% and triamcinolone 40 mg and intrathecal injections on January 21 with bupivacaine 0.5% and triamcinolone 40 mg as a part of another inpatient multimodal therapy. Previously known from a computer tomography as reason for the pain episode were intervertebral disc protrusion L2-S1 with spondylarthrosis and facet joint arthrosis as well as irritation of the nerve from L5. The maximum score according Numeric Rating Scale (NRS) was given as 8–10 (previous year NRS 7–8), the walking distance was the same at 400-500 m, and there were still no sensorimotor deficits. The patient’s home pain medication included celecoxib 2x100mg p.o. In addition, oxycodone/naloxone 2x20mg p.o. and metamizole 4x1g intravenously were administered in the hospital. Additional known comorbidities were disc protrusions L3-S1 on the left, accompanied by spondyloarthritis of the lumbar spine, arterial hypertension, epilepsy, hypothyroidism and moderate depressive episodes. Therefore, the patient was also treated with torasemide 1x5mg, bisoprolol 1x5mg, levothyroxine 1 × 125 μg, candesartan 1x16mg, amlodipine 1x5mg and valproate 2x300mg.

Shortly thereafter, the patient showed signs of septic shock which began on January 23 with hypotension and increasing infection parameters (interleukin 6: 2610 pg/ml, CRP 358 mg/l, PCT 1,79 μg/l). This was interpreted to be associated with the abovementioned injection therapy. The patient had to be transferred to the intensive care unit. There she received a catecholamine therapy (norepinephrine perfusion with up to 0.2 μg/kg/min) and calculated antibiosis with meropenem (3 × 2 g) and clindamycin (3 × 600 mg). Since the patient had progressive pain in the back, right flank and right thigh, a computer tomography examination was performed to find the cause. This examination revealed a necrotizing fasciitis - suspicious finding consisting of subcutaneous fluid and air extending from the right thigh to the right knee. The patient presented clinically in a reduced general and obese nutritional state (BMI 31) and exhibited a mild circulatory depression. She suffered from massive pain in the entire right leg and back with accompanying redness of the area descending from the back over the right hip to the right knee. Furthermore, edematous soft tissue tension was palpable ranging from the right ankle to the right shoulder.

**Fig. 1:**
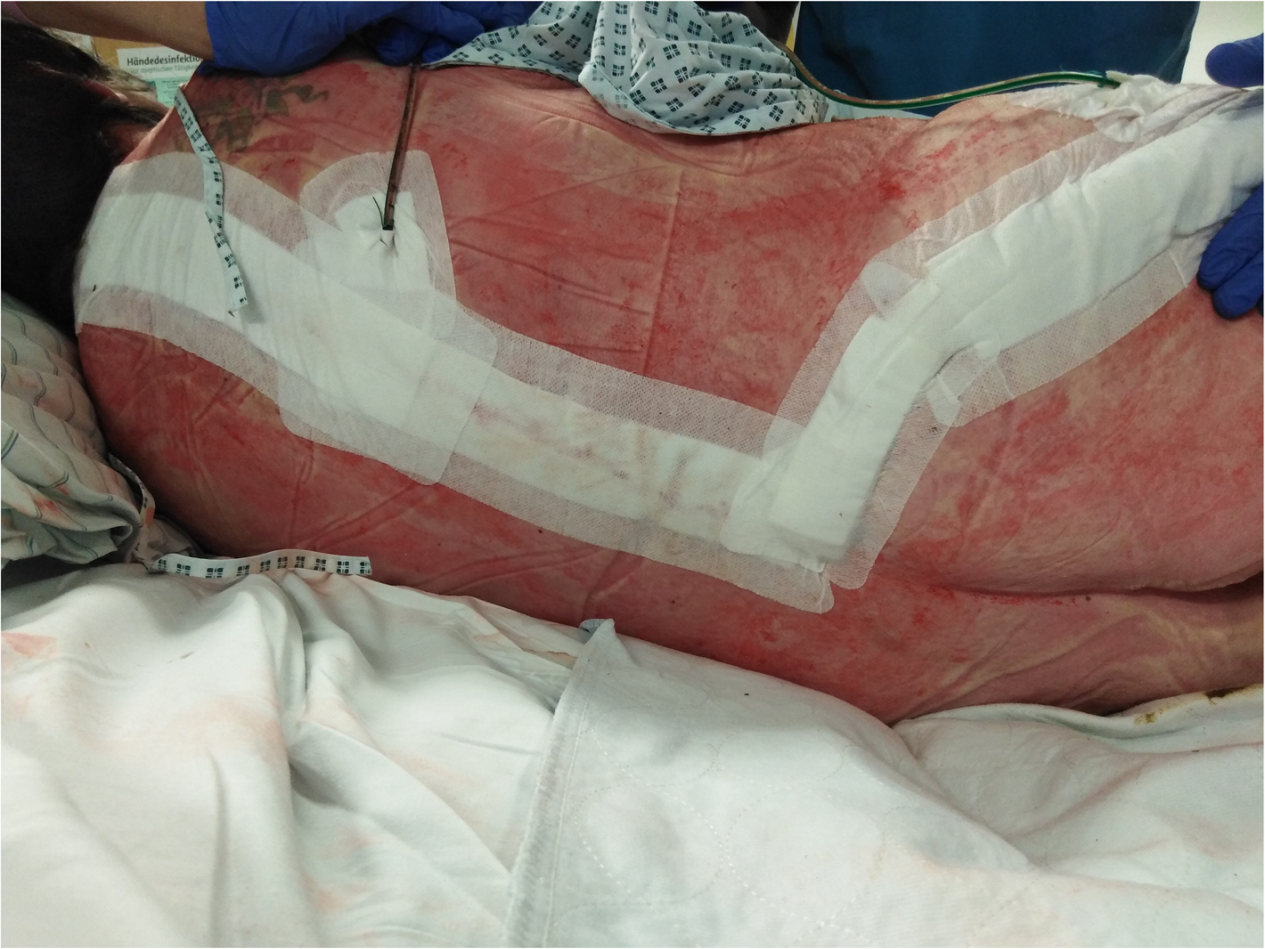
Results after multiple wound revisions and secondary wound closure

Due to the rapidly progressing findings and the prevailing circulatory conditions, the immediate emergency surgical indication for an aggressive debridement of the necrotic tissue was made. The patient received an oral explanation and was intubated and ventilated in the operating room. The clinical findings were examined under anaesthesia, followed by wound debridement and vacuum-assisted-closure therapy (VAC). The patient had to remain intubated and ventilated on the intensive care unit of our hospital. An interdisciplinary discussion of the medical staff made the indication for an adjunctive therapy with HBO. She received a paracentesis and we started immediately with HBO. HBO was carried out according to Boerema TS 300–90 (one fraction) and Marx TS 240–90 (23 fractions). We continued antibiotic therapy extended by ciprofloxacin (3x400mg), after microbiological detection of *Escherichia coli*. In the following weeks, further wound revisions and HBO therapy sessions were carried out. To complement the pain therapy, the patient received a hydromorphone PCIA with 0.5 mg boli to cope pain peaks as well as metamizole 1 g every 6 h (Patient controlled intravenous analgesia) and was supported by the pain service unit. Five weeks after admission, the patient underwent plastic surgery with a rotating plastic flap on the right lower leg to the right patella and negative microbiological swab smears (Fig. 1). The further course of the patient was without complications.

## Discussion and conclusion

There are few conservative therapy methods with a significantly positive long-term effect for the treatment of chronic non-specific back pain. For therapy-refractory patients, as described in the present case, a multimodal therapy concept is always required. Injection therapy can be used after individual consideration, but has no evidence-based proven long-term effect [8]. Based on two meta-analyses [9, 10], the use of intrathecal and lumbosacral intra-articular injections can be proposed for the treatment of chronic spinal pain and chronic low back pain. However, there are only a few studies that have systematically investigated these procedures, which, moreover, must be regarded as studies of rather limited scientific conclusiveness. In these studies, no hazardous complications such as infections were reported. For instance, Kanai et al. (2017) described the therapy of chronic lower back pain using intrathecal bupivacaine injection as safe and effective [9].

The development of necrotizing fasciitis through injection therapy is nevertheless possible and is accompanied with an extremely complicated course with high lethality [5, 10]. In addition to the injection, the patient showed other risk factors including arterial hypertension and obesity, as mentioned previously,to develop complications such as necrotizing fasciitis [5, 10, 11]. This emphasizes the importance of the individual risk-benefit assessment that must be carried out before such invasive procedures are used. Nevertheless, the timely diagnosis of necrotizing fasciitis and the transfer to a center with HBO possibility, as presented in our case, led to a cure of this potential lethal disorder.

The treatment of necrotizing fasciitis consists primarily of early and aggressive surgical treatment as well as accompanying antibiotic therapy and supportive care. It was reported that a treatment delay (mortality from intervention within 24 h 6%, between 24 and 48 h 24%), of the operative care as well as an insufficient surgical debridement contribute to an increase in the mortality rate [6]. There are only few reported experimental data for HBO therapy in necrotizing fasciitis, although clostridial gangrene is considered well studied with this therapy. Nonetheless, a reduction in mortality and morbidity for necrotizing fasciitis with HBO is suggested based upon results from smaller studies [6, 11].

In conclusion the therapy of chronic non-specific back pain continues to be a challenge for doctors and patients. As part of the multimodal pain concept, invasive therapy measures should be discussed and implemented individually. When using injection therapies, these must be carried out strictly aseptically. The development of pain after injections should be accompanied by an immediate consultation with the attending physician in order to be able to identify and treat serious life-threatening, such as necrotizing fasciitis.

The mainstays of therapy of a necrotizing fasciitis include early and aggressive surgical debridement, antibiotics and supportive care. Adjuvant methods such as protein synthesis inhibitors, hyperbaric oxygen and intravenous immunoglobulin may play a role in the treatment, but further proof of efficacy is necessary to allow for an evidence-based recommendation.

## Data Availability

The datasets generated and analysed for the case report are not publicly available due to protect participant confidentiality. They are available from the corresponding author on reasonable request.

## Abbreviations

BMI: Body-Mass-Index
HBO: Hyperbaric oxygenation
NRS: Numeric Rating Scale
ISG: Sacroiliac joint
VAC: Vacuum-assisted-closure therapy
